# Editorial: The Mechanisms of Action of Anti-SARS-CoV-2 Drugs

**DOI:** 10.3389/fphar.2022.894310

**Published:** 2022-04-04

**Authors:** Wu Zhong, Vincenzo Brancaleone, Janet Sultana

**Affiliations:** ^1^ National Engineering Research Center for the Emergency Drug, Beijing Institute of Pharmacology and Toxicology, Beijing, China; ^2^ Department of Science, University of Basilicata, Potenza, Italy; ^3^ College of Medicine and Health, University of Exeter, Exeter, United Kingdom

**Keywords:** small-molecule drug, SARS-CoV-2, drug repurposing, mechanism of action, target-based drug design

The coronavirus disease 2019 (COVID-19) pandemic has brought great uncertainty to global economic development and social stability and posed a great threat to human life and health. The most effective response to the pandemic remains a combination of “vaccines plus drugs.” The success of severe acute respiratory syndrome coronavirus 2 (SARS-CoV-2) vaccines has reduced the morbidity, severe illness and mortality caused by COVID-19 and, to a certain extent, has curbed the global spread of SARS-CoV-2. However, the emergence of iterations of SARS-CoV-2 variants has led to a reduction in the protection afforded by the marketed vaccines and even frequent breaches in the immune barrier. Although the Food and Drug Administration (FDA) has urgently authorized the use of several antibodies as drugs for COVID-19 treatment, these molecules still show significant limitations. First, they can be used only to treat patients with mild-to-moderate disease and be only administered intravenously, which is costly and limits their widespread use. Second, the mechanisms of action of antibody drugs predictably cause drug resistance problems, and none of the approved antibodies are effective against the currently prevalent *omicron* variant. In combating COVID-19, orally available small-molecule drugs are indispensable and may be the centerpiece in the prevention and control of the pandemic. Small-molecule drugs have shown excellent clinical effects in pre- and postexposure prophylaxis as well as in the early treatment of severe disease. Thus, the availability of these anti-SARS-CoV-2 drugs will turn COVID-19 into a preventable and treatable disease and provide an extremely important assurance for us to win the fight against SARS-CoV-2, therefore leading to elimination of the requirement for masks and a return to normal daily life in the near future.

Small-molecule anti-SARS-CoV-2 drugs can be taken orally, mass produced, and supplied in large quantities with good accessibility and affordability. Especially since a large number of COVID-19 patients are currently mild-to-moderately ill, these patients can be treated at home with small-molecule drugs, which can greatly reduce the pressure on health care resources and the risk of cross-contamination. More importantly, these small-molecule drugs are targeted at relatively conserved sites of the virus to overcome drug resistance, and they maintain activity against SARS-CoV-2 variants of concern. All these advantages make small-molecule drugs unparalleled in COVID-19 treatment and the prevention and control of the pandemic.

Drug repurposing is the most efficient drug discovery strategy in response to an unexpected emerging infectious disease in the early stage of the outbreak. Global efforts in small-molecule drug discovery have mainly focused on screening of drugs approved by the FDA or in the clinical stage, such as favipiravir, remdesivir, ebselen, and masitinib, to accelerate the evaluation of such drugs for COVID-19 by considering both their human pharmacokinetic properties and toxicity. Since the pandemic began, drugs that have advanced into clinical studies through drug repurposing strategies have dominated, and the most successful drug is remdesivir. Remdesivir is the only FDA-approved drug for COVID-19 treatment to date. However, due to the severity of the pandemic in the early stage, many of the clinical studies conducted did not follow the standard randomized control trial (RCT) design, resulting in low recognition of drug effectiveness.

With the in-depth understanding and scientific knowledge of the viral infection process and pathogenesis, rational design for viral and host targets has become a more effective strategy to develop anti-SARS-CoV-2 drugs. Therefore, the elucidation of the mechanism of action of candidate anti-SARS-CoV-2 molecules is the keystone to evolve toward broad-spectrum and target-specific drugs. In this research topic, we focus on the mechanism of action of anti-SARS-CoV-2 drugs, with the aim of promoting the development of precision drugs based on rational design. At present, the targets for the development of anti-SARS-CoV-2 small-molecule drugs are mainly focused on viral RdRp and 3CLpro. Drugs developed based on this strategy include but are not limited to molnupiravir and vv116 targeting viral RdRp and PF-07321332, s-217622, and FB2001 targeting viral 3CLpro (Zhao et al.). ACE2 is a major receptor for SARS-CoV-2 infection and an important target for drug design, and its expression level correlates with the level of immunity in the body after infection. The ACE2 level is regulated by multiple ISGs and is highly correlated with its induced NF-κB pathway activity (Yan et al.). A clinical study showed that genotype-specific cytokine storms and immune responses were enriched in patients with ACE deletion polymorphisms, suggesting that polymorphisms in the ACE gene may be associated with susceptibility to the novel coronavirus (Gong et al.). All these studies provide references for precise drug design. The elucidation of the drug mechanism of action also provides a theoretical basis for the synergistic use of drugs to improve their efficacy while reducing drug resistance.

With the in-depth research by scientists worldwide on viral pathogenesis and drug mechanisms of action, as well as the experience accumulated by the pharmaceutical industry in various countries over many years, an end to the pandemic may come soon. Small-molecule oral antivirals may be the “space-time artifact,” allowing people to return to normal daily life as it was back in 2019. Given the potential RNA viruses to cause pandemics due to their characteristics of rapid variation, the development of broad-spectrum antiviral drugs with multiple targets should be prioritized. Therefore, a relevant small-molecule drug library should be established for the common targets of RNA viruses to improve the ability to respond to possible outbreaks of new emerging infectious diseases in the future.

