# Corrigendum: Editorial: The mechanisms of action of anti-SARS-CoV-2 drugs

**DOI:** 10.3389/fphar.2022.1065930

**Published:** 2022-11-07

**Authors:** Wu Zhong, Vincenzo Brancaleone, Janet Sultana

**Affiliations:** ^1^ National Engineering Research Center for the Emergency Drug, Beijing Institute of Pharmacology and Toxicology, Beijing, China; ^2^ Department of Science, University of Basilicata, Potenza, Italy; ^3^ College of Medicine and Health, University of Exeter, Exeter, United Kingdom

**Keywords:** SARS-CoV-2, drug repurposing, mechanism of action, small-molecule drug, Target-based drug design

Authors “Vincenzo Brancaleone and Janet Sultana” were not included as authors in the published article.

The corrected Author Contributions Statement appears below.

“WZ completed the draft manuscript, VB and JS contributed to the drafting of the paper and approved the final version.”

The authors apologize for this error and state that this does not change the scientific conclusions of the article in any way. The original article has been updated.

